# Can oral health care be a gateway to improve cardiovascular disease? A scoping review

**DOI:** 10.3389/froh.2024.1364765

**Published:** 2024-05-22

**Authors:** Wania Usmani, Maximilian de Courten, Fahad Hanna

**Affiliations:** ^1^ Department of Health, Torrens University Australia, Melbourne, VIC, Australia; ^2^ Health and Education Policy, Mitchell Institute, Victoria University, Melbourne, VIC, Australia; ^3^ Public Health Program, Department of Health and Education, Torrens University Australia, Melbourne, VIC, Australia

**Keywords:** oral health care, periodontitis, oral hygiene care, periodontal disease, oral health promotion, cardiovascular diseases, cardiovascular severity

## Abstract

**Background:**

Cardiovascular diseases (CVDs) are a significant cause of morbidity and mortality worldwide, resulting in a high socioeconomic burden. Growing evidence has shown a link between oral diseases and several chronic conditions including CVDs. The focus of this review is to investigate and summaries the evidence surrounding oral health interventions and their potential impact on reducing both the risk and/or severity of CVDs.

**Methods:**

A scoping review was conducted to examine oral health interventions for managing CVD outcomes and risks. The review adhered to the Joanna Briggs Institute (JBI) framework for evidence synthesis and followed the reporting standards outlined by the Preferred Reporting Items for Systematic Reviews and Meta-analysis- extension to Scoping Review (PRISMA-ScR). A systematic search across EBSCOhost, PubMed, and Scopus databases from 2012 to 2024 was utilized to identify relevant studies. Inclusion criteria focused on English language articles with a sample size of at least 50, evaluating the impact of oral health interventions on CVD outcomes.

**Results:**

Out of the initial 2,154 studies identified in the search, 12 studies met the inclusion and exclusion criteria and were included in the final analysis. Overall, the studies revealed that along with surgical and non-surgical periodontal therapy, regular oral hygiene care practices, including toothbrushing, tongue brushing, and flossing, significantly reduced the risk of cardiovascular events and mortality. These interventions in patients with or without CVD baseline have shown a decrease in CVD risk markers as well as a reduction in bacterial colonization. Similarly, consistent oral hygiene routines, combined with regular dental visits, were associated with a lower risk of heart failure and CVD risk mortality.

**Conclusion:**

The evidence extracted from this review suggests that periodontal therapy, regular dental cleaning, and re-enforcing of oral health regimes can stabilize oral health conditions and subsequently improve CVD progression/risks. However, limited to no evidence exists regarding the therapeutic effects of oral health promotion in managing CVD markers and its direct impact on disease outcomes, warranting further investigation.

## 1 Introduction

Cardiovascular diseases (CVDs) pose a considerable global health burden, contributing to a significant number of deaths each year according to the World Health Organization (1). While traditional risk factors for CVDs, such as hypertension and hyperlipidemia, are widely recognized, emerging evidence points to a robust connection between oral health and cardiovascular health. Given this evolving understanding, the focus of this review is to investigate the evidence surrounding oral health interventions and their potential impact on reducing both the risk and/or severity of CVDs.

Likewise, the burden of oral health diseases is a significant public health concern as it extends beyond physical health implications, impacting an individual's quality of life, social interactions, and economic productivity, resulting in low self-esteem and risk of social isolation (1). From 2011 to 2020, approximately 62% of dentate adults were estimated to have periodontitis, with severe cases accounting for 23.6% (2). Additionally, WHO estimates that 2 billion individuals suffer from dental caries in permanent teeth, while 514 million children suffer from caries in primary teeth (3). A plethora of evidence suggests that oral health diseases are linked to higher rates of mortality from the four most prevalent non-communicable diseases (NCDs) including CVDs, cancer, diabetes, and chronic respiratory diseases (4–8). Despite the significant focus on NCD prevention, detection, and treatment over recent decades, oral health care has been overlooked by public health systems (3); Nguyen et al., (9).

The microbiome of the oral cavity is the second largest and most diverse microbiota after the gut, harboring over 700 species of bacteria as well as fungi, viruses, and protozoa (10). The ecological niches in the oral cavity are divided into the saliva, tongue, dental surface, gingiva, buccal mucosa, palate, and subgingival/supragingival sites, with variations in species but dominated by Streptococcus, which produces an abundance of primary and secondary metabolites and (11). In recent years, an impressive number of studies have shown that these bacteria play a leading role in the proper functioning of the metabolism and the immune system, however, when imbalances in the composition of the oral microbiome occur as a result from dental caries or periodontal disease, the bacterial proliferation can lead to the development or severity of several chronic diseases, specially cardiovascular diseases (11–14).

### 1.2 Oral health’s impact on heart health: unraveling connections

In the oral cavity, periodontal diseases and dental caries disrupt the oral microbiome, resulting in oral dysbiosis, leading to the exacerbation of various systemic diseases, including CVDs (13, 15). Dental caries, characterized by non-communicable, net-mineral loss of dental hard tissues, results from acid production by cariogenic bacteria fermenting free sugars (16–18). This oral health condition fueled by fermentable sugars, initiate a cascade of inflammatory responses within the oral cavity which results in increased production of an acute-phase protein known as C-reactive protein (CRP) (19). Increased CRP level is considered as a risk factor for developing cardiovascular diseases (20, 21) and hypertension (20, 22). Concurrently, oral bacteria translocate into the bloodstream from dental carious lesions, infiltrating arterial walls and triggering an inflammatory response, ultimately contributing to atherosclerotic plaque formation and cardiovascular complications (23).

Similarly, periodontitis, a chronic inflammatory form of periodontal disease affecting the supporting structures of the teeth, increases the magnitude of oral dysbiosis and serves as a potent contributor to CVD risk (4, 12). Poor oral hygiene serves as the primary cause of periodontitis, with social determinants of health such as income and education level, and poor lifestyle choices influencing periodontal outcomes (24). Severe periodontitis leads to tooth loss, which has been identified as a marker for increased risk of heart failure (HF) and hospitalization for various types of CVD, including ischaemic heart disease (IHD), and peripheral vascular disease (PVD) (25–27).

**Figure 1:**
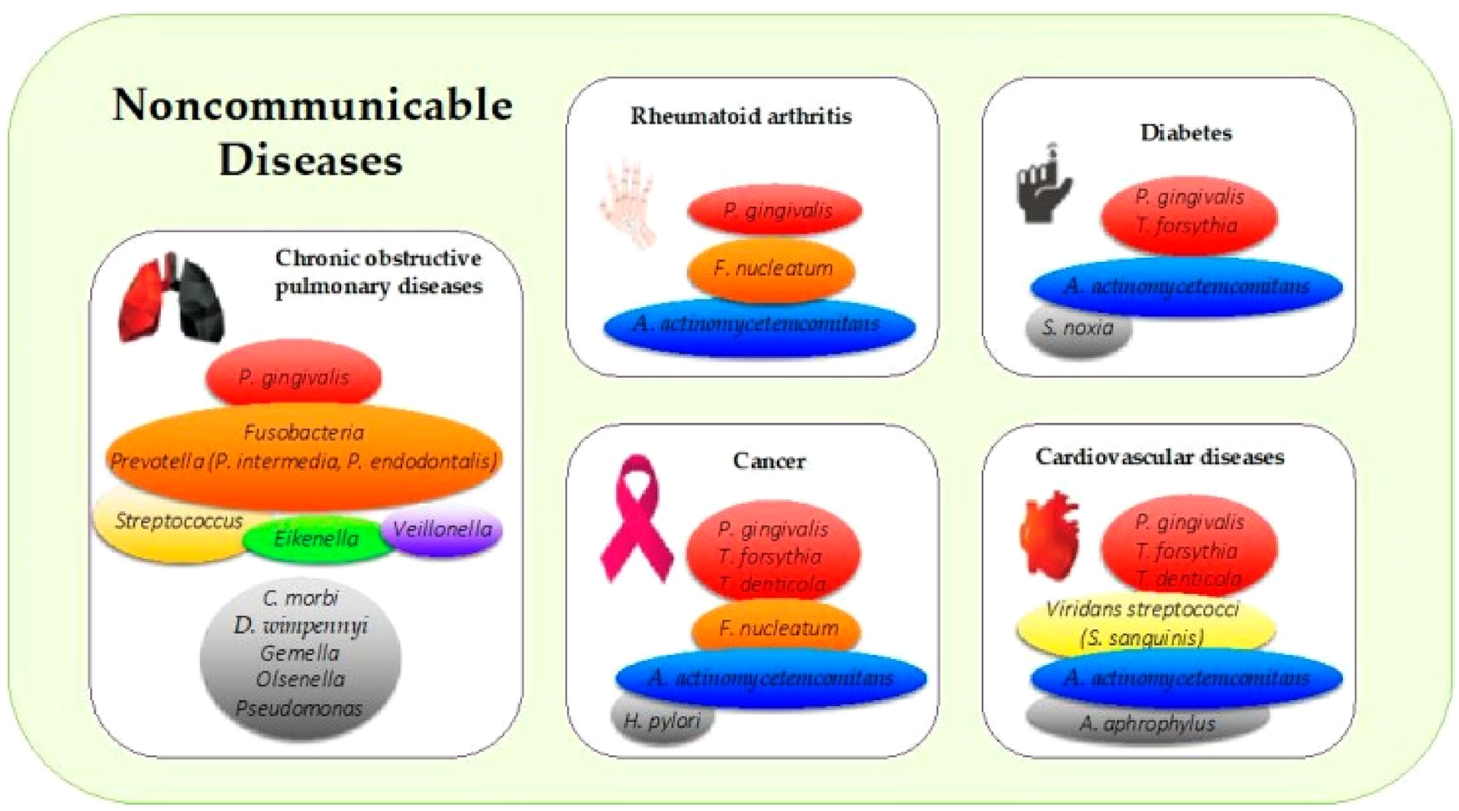
Periodontal pathogens implicated in the most common noncommunicable diseases. The colors in boxes refer to (i) the colors of the Socransky complexes for the purple, green, yellow, orange, and red colors, and (ii) other periodontal bacteria for the gray color. A. actinomycetemcomitans: Aggregatibacter actinomycetemcomitans; C. morbi: Cantonella morbi; D. wimpennyi: Dysgonomonas wimpennyi; F. nucleatum: Fusobacterium nucleatum; H. pylori: Helicobacter pylori; P. gingivalis: Porphyromonas gingivalis; S. noxia: Selenomonas noxia; S. sanguinis: Streptococcus sanguinis; T. denticola: Treponema denticola; T. forsythia: Tannerella forsythia (4).

Some periodontal pathogens (see Figure 1) may exacerbate periodontitis induced-inflammation and bacteremia and, contributing to clogging of arteries by accumulating monocytes and macrophages in arterial walls, impeding blood vessel relaxation and dilation, and increasing arterial stiffness (4). These bacterial and inflammatory mediators disseminating from the oral cavity to the bloodstream induce endothelial dysfunction, inflammation, oxidative stress, and atherosclerotic plaque formation which can lead to an increased risk of cardiac diseases such as heart failure, artherosclerotic cardiovascular diseases (ACVDs), stroke and possibly other serious CVD complications (23, 27, 28). The risk of developing serious CVD complications from periodontitis-induced bacteremia, varies among individuals due to a combination of factors including overall health, immune response, genetic disposition, and lifestyle choices such as diet and smoking habits (14, 23, 29). While bacteremia can occur in anyone, those with weakened immune systems or underlying health conditions may be more susceptible to CVD complications. Additionally, the type and virulence of the bacteria involved can influence the severity of CVD outcomes (4). On the other hand, A prospective study by Donders et al. (30) argues that while periodontitis is not an independent risk of CVD severity and risk, it is associated with increased CVD risk and can serve as an important link to bridge the gap between dentistry and general medicine to identify patients at risk for CVD in an earlier stage. The aim of this scoping review was to investigate whether oral health interventions can reduce CVD risk and/or severity.

## 2 Methodology

A scoping review of the literature was conducted to assess oral health interventions to improve/manage CVD outcomes and risks of CVDs. This approach was considered as it allows a wider exploration of the subject matter while maintaining the credibility and transparency of existing knowledge (31). The review methodology followed Joanna Briggs Institute's (JBI) framework for evidence synthesis (32) and complied with the reporting standards set by the “Preferred Reporting Items for Systematic Reviews and Meta-Analyses-Extension for Scoping Reviews (PRISMA-ScR)” (33).

### 2.1 Search strategy

A systematic database search of articles published in the last 12 years (2012–2024) and related to oral health interventions and CVD outcomes was performed. The search was carried out using the EBSCOhost, PubMed and Scopus databases using this search term: (“oral health care” OR “dental care” OR “periodontal disease” OR “gingivitis” OR “periodontitis” OR “dental hygiene” OR “oral hygiene”) AND (“cardiovascular diseases” OR “heart diseases” OR “coronary artery disease” OR “myocardial infarction” OR “stroke” OR “cardiovascular risk” OR ‘cardiovascular severity) AND (“intervention” OR “management” OR “treatment” OR “prevention”).

### 2.2 Eligibility criteria

To screen the best-suited articles, inclusion criteria focused on primary studies that explored the relationship between oral health care and cardiac disease management, had been published between 2012 and 2024, and were available in English and in full text or belonging to journals accessible via Torrens University Australia library. Studies were only included if they evaluated the impact of oral health interventions (e.g., periodontal treatment, dental hygiene or dental scaling, oral health education) on CVD outcomes (e.g., incidence, severity), and had a sample size of 50 and above. To ensure reliable level of evidence, this review evaluated prospective cohort studies, clinical trials and case-control studies.

### 2.3 Articles selection process

**Figure 2:**
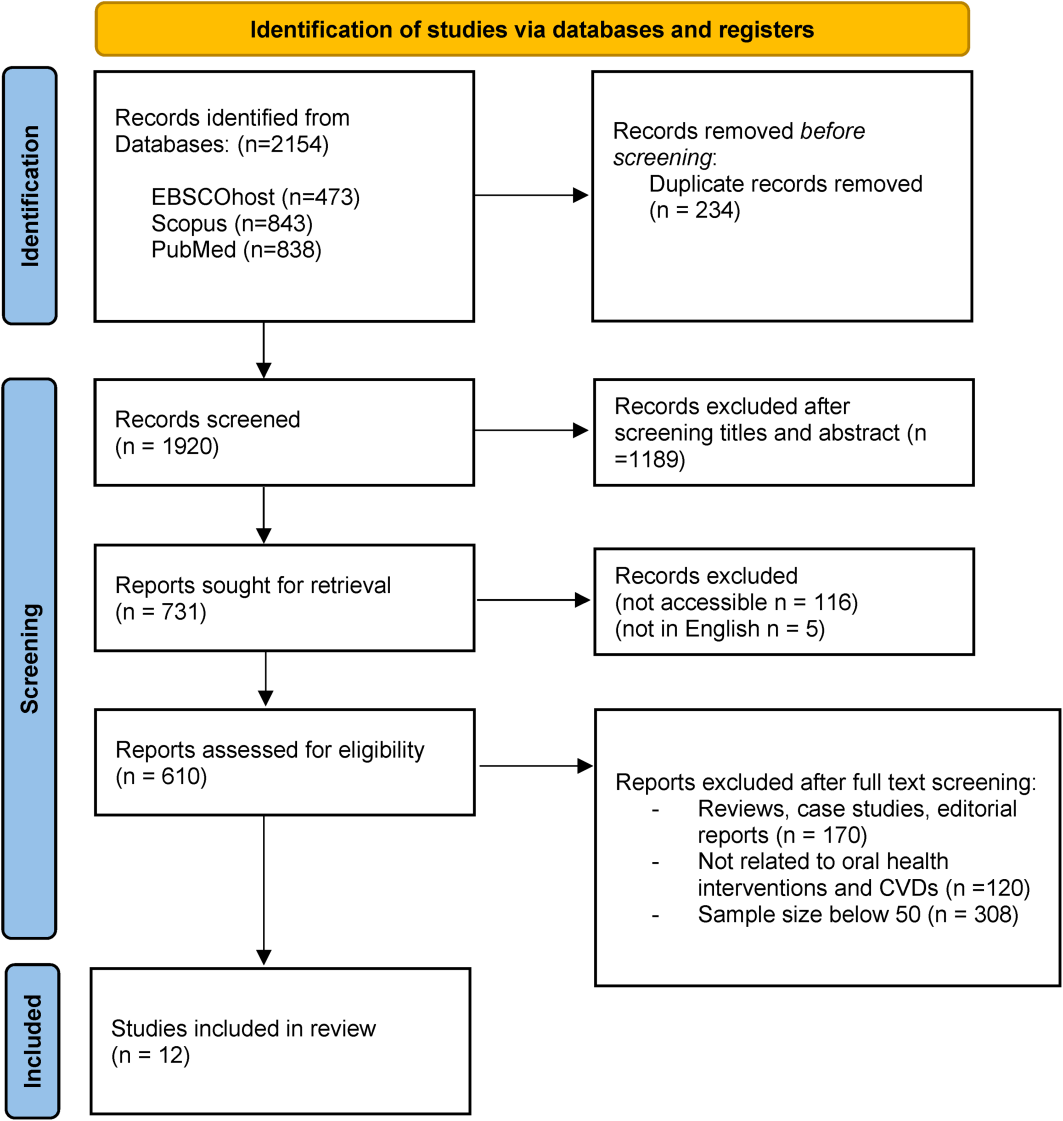
PRISMA flow diagram for scoping review (57).

The initial systematic bibliographic database searches retrieved a total of 2,154 records. After excluding 234 duplicates and another 1,189 records due to ineligibility/irrelevance upon screening of titles and abstracts, 121 articles were further excluded due to inaccessibility and language issues. The remaining 731 articles underwent full-text assessment to determine their eligibility for inclusion in the review. As a result, 719 Ineligible articles were further excluded from the review. Reasons for exclusion were documented (see Figure 2). A total of 12 articles were selected as ‘final studies’ which have been documented, along with their characteristics and key findings.

## 3 Results

### 3.1 study characteristics

The scoping review encompasses a diverse array of studies conducted in various global contexts, reflecting a broad spectrum of methodological approaches and interventions aimed at reducing CVD risk markers and outcomes by addressing periodontal health and oral hygiene care. Characteristics of the included 12 studies are summarized in Table 1.

**Table 1:** Study characteristics and results table.

Author/year/study	Study design	Sample size	Oral health intervention	CVD outcomes/ CVD risk markers
(34), Pakistan	Randomised control trial	246	Periodontal therapy (non-surgical)	Significantly reduced levels of C-reactive protein, fibrinogen and white blood cells
(35), Brazil	Randomised control trial	66	Periodontal therapy (non-surgical)	Significant reduction in inflammatory markers [C-reactive protein, erythrocyte sedimentation rate (ESR), total cholesterol, and triglycerides]
(40), India	Case-control study	150 (50 healthy control, 50 with chronic periodontitis and 50 with aggressive periodontitis)	Periodontal therapy (surgical)	Significant decrease in C-reactive proteins
(41), Netherlands	Case control and pilot intervention study	105 (57 periodontitis patients and 48 healthy control)	Periodontal therapy (non-surgical)	No changes in pulse-wave velocity (PWV). Significant reductions in systolic blood pressure, diastolic blood pressure, total cholestrol, high-density lipoproteins and low-density lipoproteins
(39), China	Randomised control trial	107 patients with prehypertension and periodontitis	Intensive periodontal treatment (surgical periodontal therapy)	Immediate reduction in systolic blood pressure. Significant decrease in diastolic BP, endothelial microparticles and C-reactive proteins after 3 and 6 months but reduction in interleukin-6 levels after 6 months of treatment
(38), Brazil	Randomised control Trial	69 patients with stable coronary disease and severe periodontitis	Periodontal therapy (non-surgical	No changes in FMD (flow mediated dilation)—marker of vasodilation. Significant reduction in soluble cell adhesion molecules (sVCAM-1, sICAM-1, and P-selectin)—markers of vascular inflammation
(37), Brazil	Randomised control trial	82	Periodontal therapy (surgical)	Significant reduction in C-reactive protein, interleukin-8 and interleukin-8 levels
(36), USA.	Randomised control trial	51 post-stroke survivors	Oral Hygiene Care Protocol (tooth brushing, tongue brushing, flossing, mouth rinse, and lip care)	Statistically non-significant overall reductions in methicillin-resistant Staphylococcus aureus and methicillin-sensitive Staphylococcus aureus colonization in the intervention group
(50), Korea	Population based observational study; cohort study	247,696 healthy adults (with no history of major CVD events)	Oral hygiene care (Regular dental visits and frequent brushing	Brushing twice or more/day significantly lower risk of cardiovascular events by 9%. Regular dental visits (once a year or more) for professional cleaning significantly lowers the risk of cardiovascular events by 14%
(42), Korea	Longitudinal Cohort Study	173,927 patients with type 2 diabetes	Combined oral hygiene care (professional cleaning and toothbrushing)	Brushing more than once was decreased risk of Heart Failure (HF). One or more dental visits decreased risk of HF. Combined oral hygiene care (professional cleaning and brushing twice daily) showed further significant HF risk reduction
(43), USA	Longitudnal cohort study	354 dentate participants OHS	OHS: Oral hygiene self-care (brushing, flossing and mouthwash use)	Marginally significant CVD mortality risk reduction in coronary heart disease patients. 51% CVD mortality risk reduction in healthy dentate patients. No effect of mouthwash on OHS
(45), Turkey and Denmark Turkey	Stratified prospective sttudy	(TR) = 186 Denmark (DR) = 116	Health Promotion (Health Coaching Model)	reductions in Hb1AC and periodontal index in health coaching group

Six studies were randomised control trials (RCTs) (34–39), 2 studies were case-control studies (40, 41), 3 studies were cohort studies (42–44) and 1 was a stratified prospective study (45). The geographical locations of these studies include Pakistan (34), Brazil (35, 37, 38) and China (39), India (40), Korea (42, 44), Netherlands (41), USA (36, 43), Turkey and Denmark (45). The included studies had no restrictions on age, gender and socioeconomic status. These studies examined the efficacy of non-surgical periodontal therapies (characterized by supragingival and subgingival scaling and root surface debridement) and surgical periodontal therapies (characterized by guided tissue regeneration achieved by re-contouring of gum and bone), oral hygiene care and oral health promotion across diverse geographical groups.

### 3.2 Key themes

#### 3.2.1 Periodontal therapy (PT)

The overall findings from seven studies investigating the effectiveness of periodontal therapy (PT) on cardiovascular health outcomes showed a protective effect of PT against CVD risk markers. A randomized controlled trial in Pakistan (34) demonstrated that non-surgical PT led to reduced levels of C-reactive protein, fibrinogen, and white blood cells among 246 participants. Similarly, in Brazil, another randomized controlled trial with 66 participants (35) found that non-surgical PT resulted in a reduction in inflammatory markers, including C-reactive protein, erythrocyte sedimentation rate (ESR), total cholesterol, and triglycerides. Additionally, a case-control study in India (40) involving 150 participants revealed a decrease in C-reactive proteins following surgical PT. Furthermore, studies conducted in the Netherlands, China, and Brazil showed improvements in various cardiovascular risk factors such as blood pressure, lipid profile, and inflammatory markers following both non-surgical (38, 41) and surgical (37, 39) PT interventions.

#### 3.2.2 Oral hygiene care

Four studies highlighted the importance of maintaining good oral hygiene (36, 42–44). RCT conducted among post-stroke nursing home patients compared oral care twice daily in the intervention group, this oral care protocol included toothbrushing, tongue brushing, flossing, mouth rinse, and lip care, with standard oral care (brushing only) once daily in the control group. Results showed almost double the colonization of Staphylococcus aureus in the control group compared to the intervention group. In the intervention group, the bacterial load was reduced from 20.8% to 16.7% (36). Similarly, a nationwide trial among healthy adults (≥40 years) without baseline CVDs reported a 9% reduction in fatal/nonfatal cardiovascular events for every unit increase in daily toothbrushing frequency over a median follow-up of 9.5 years. This reduction was more pronounced when toothbrushing was combined with regular professional oral hygiene attendance, resulting in a 14% risk reduction (44). A recently conducted cohort study on type 2 diabetes patients in Korea (42) observed similar findings where brushing teeth two times daily and attending one or more dental visits were linked to lower HF risk. Combining these oral hygiene practices further reduced the risk of HF (*P *= 0.024). Additionally, Oral hygiene self-care (OHS) (twice/day brushing, regular flossing and mouthwash usage) was also studied in 256 patients with and without confirmed diagnosis of coronary artery disease (43) and reported a significant correlation between better oral hygiene (brushing with flossing) and reduced the risk of CVD mortality by 51% (HR 0.49, *p* = 0.01), those who had coronary artery disease at baseline showed a marginally significant benefit (HR 0.50, *p* = 0.07). However, mouthwash usage did not have any effect on OHS (HR = 0.49, *p* = 0.01), indicating no additional benefits nor detriments during the 18.8 years of follow-up.

#### 3.2.3 Oral health promotion/education

Only one study (45) explored effect of oral health promotion on managing diabetes, a potential risk factor for CVD development and/or progression. The clinical trial by (45) adapted the Health Coaching Model to help diabetic patients develop a vision and identify the connection between oral health care and diabetes management by conducting 20–60 min 3–4 face-to-face sessions of oral health coaching, the results from this coaching significantly enhanced patients’ commitment to change and showed a reduction of HbA1c and improved oral health parameters in the health coaching group compared to control group (TR: 0.8%; DK: 0.4%, *P* < 0.01)

## 4 Discussion

The literature identified in this review confirms a strong link between oral health and cardiac health by addressing the impact of oral health interventions; PT (surgical or non-surgical), oral hygiene care (tooth brushing, flossing and professional dental care) on CVD outcomes. In support of these findings, a systematic review with meta-analysis published in 2018 concluded that periodontal treatment has a beneficial effect on some of the biochemical parameters considered to be representative of cardiovascular risk (46), however, even with the positive results recorded in this systematic review, authors emphasized the need for more homogeneous clinical trials, with larger samples, more detailed methodology and longer follow-up periods to generalize these findings (46). While the number of comprehensive clinical trials specifically evaluating the effects of oral health interventions on significant cardiovascular outcomes is limited, the underlying mechanisms by which oral health may mitigate cardiovascular disease risk are evident.

Despite progress in medical and oral care and efforts to improve interdisciplinary coordination and collaboration in healthcare, few findings from this review emphasize the ongoing lack of awareness of the link between oral health and chronic diseases, particularly among vulnerable groups such as pregnant women and those susceptible to systemic conditions like diabetes and hypertension. In light of growing evidence on oral health care and its impact on CVDs, many of the high-risk cardiac patients and even cardiac- clinicians lack awareness/knowledge of this association which often leads to ignorance among clinicians to provide oral health assessment, education, and dental referrals for patients with CVD (47, 48). Oral health promotion is an under-looked yet effective way to increase one's awareness of oral health and its association with systematic conditions, and empower individuals across all age groups to take control of their oral health and potential their risk of systemic conditions (49). The primary objective of oral health education programs is to enhance the oral health of individuals by enhancing their oral health care behavior. Some studies in this review have demonstrated the efficacy of such programs in both children and adults, with and without chronic conditions, with such programs proving to be successful in improving knowledge and attitudes toward dental care, thereby reducing the prevalence of oral diseases (50–52). Oral health education for increasing oral health awareness and care among CVD patients has not yet been fully explored. This review identified one solitary study that explored the effectiveness of oral health promotion by health coaching model on improving oral health and Hb1AC levels in diabetic patients (45). Despite thelimited evidence on oral health education for CVD management, some educational models such as health coaching, motivational interviewing (51, 53) and health belief education models (52, 54) have been shown to improve oral health behaviours/awareness in primary and tertiary care to manage oral-systemic conditions.

It is widely recognized that oral diseases and systemic NCDs are interconnected through molecular and immunological pathways, sharing common risk factors and social determinants of health (55). Rather than solely focusing on disease-specific approaches to prevent and manage NCDs such as CVDs, there is a compelling argument for adopting a more comprehensive approach that considers factors beyond the healthcare system, such as socioeconomic conditions, dietary habits, demographic trends, and other external influences on health and well-being (56). Given the multifactorial nature of most chronic diseases, it is crucial to take integrated action against risk factors that contribute to various diseases. Therefore, improving poor oral health is a crucial ‘risk marker’ and warrants investigation into its effectiveness in preventing and managing NCDs, including CVDs (25).

## 5 Recommendations

This review identifies a paucity of research of the effectiveness of behavioural or oral health promotion interventions in improving oral health and oral hygiene awareness and associated cardiac disease outcomes. Such programs could be incorporated in future preventative oral health and CVD management protocols. Additionally, a systematic reform of oral health education is warranted to help educate and empower the public against oral health disease and associated risks of systematic conditions. It is highly recommended that prospective studies and trials with larger sample sizes and extended observation periods are conducted to present more reliable evidence on the impact of oral health promotions and programs on CVD severity and management, including an assessment of whether reducing inflammatory markers through improving oral health care results in a protective effect against CVD risk and development.

## 6 Conclusion

The reviewed literature highlights the significant link between oral health and cardiovascular health, demonstrating the potential impact of oral health interventions on future cardiovascular outcomes and mortality. While promising, more comprehensive and rigorous clinical trials are needed to confirm these findings and establish stronger connections between oral health and cardiovascular outcomes. The lack of awareness regarding the relationship between oral health and chronic conditions, particularly among high-risk groups and clinicians, underscores the necessity for increased oral health education and promotion. Addressing this gap could potentially enhance patient care and potentially reduce cardiovascular disease risks. An integrated approach that considers socioeconomic and lifestyle factors alongside healthcare interventions is crucial in managing and preventing chronic diseases, including cardiovascular disease. Therefore, improving oral health through effective interventions and education should be a priority in efforts to manage and prevent NCDs.
